# Secondary Hemophagocytic Lymphohistiocytosis Presenting As the Initial Manifestation of Diffuse Gastric Signet Ring Cell Adenocarcinoma: A Case Report

**DOI:** 10.7759/cureus.107904

**Published:** 2026-04-28

**Authors:** Joana N Santos, Francisca S Batista, Beatriz Castanheira, J. Guilherme Gonçalves-Nobre, Mariana Santiago

**Affiliations:** 1 Medical Oncology, Unidade Local de Saúde de Almada-Seixal, E.P.E., Almada, PRT; 2 Neuroradiology, Unidade Local de Saúde de Almada-Seixal, E.P.E., Almada, PRT

**Keywords:** anakinra, bone marrow infiltration, gastric adenocarcinoma, hemophagocytic lymphohistiocytosis, paraneoplastic syndrome, secondary hlh, signet ring cell carcinoma, virchow node

## Abstract

Hemophagocytic lymphohistiocytosis (HLH) is a life-threatening hyperinflammatory syndrome driven by uncontrolled immune activation, most commonly associated with hematologic malignancies. Its occurrence as the presenting manifestation of a solid tumor is rare and frequently underrecognized due to significant clinical overlap with severe infectious syndromes. We report the case of a 38-year-old male with no prior medical history who presented with persistent fever, severe lower back pain, and night sweats. These nonspecific constitutional symptoms, including fever, weight loss, and night sweats, initially raised suspicion for an infectious or inflammatory etiology. Laboratory evaluation revealed severe anemia, thrombocytopenia, marked hyperferritinemia, elevated lactate dehydrogenase, hepatic cytolysis, and hypertriglyceridemia. Peripheral blood smear demonstrated a leukoerythroblastic pattern with schistocytes and circulating erythroblasts, suggestive of bone marrow infiltration. An HScore indicated an 80-88% probability of HLH, supporting a high likelihood of reactive HLH in the adult setting. Extensive infectious workup was negative, including blood and urine cultures, which remained sterile throughout hospitalization. Imaging identified a left supraclavicular lymph node conglomerate consistent with Virchow’s node and diffuse bone marrow infiltration throughout the spine, with anterior epidural extension at the D6 level. Initial computed tomography of the chest, abdomen, and pelvis did not reveal a clear primary intra-abdominal lesion but demonstrated hepatomegaly and lymphadenopathy, prompting further investigation. Upper gastrointestinal endoscopy revealed multiple large gastric ulcers, and histopathological analysis confirmed diffuse gastric adenocarcinoma with signet ring cell features. The tumor demonstrated proficient mismatch repair (pMMR), PD-L1 combined positive score (CPS) 5, HER2-negative status, and claudin 18 (CLDN18) positivity. A bone marrow biopsy was non-diagnostic due to extensive necrosis but showed immunohistochemical evidence suggestive of metastatic epithelial infiltration. Immunomodulatory therapy with anakinra was initiated; however, persistent severe thrombocytopenia precluded systemic oncologic therapy. The patient died 28 days after admission from multiorgan failure, highlighting the fulminant course and high mortality associated with malignancy-triggered HLH. This case highlights the importance of early consideration of malignancy in unexplained HLH and illustrates how hematologic compromise may preclude disease-modifying treatment.

## Introduction

Hemophagocytic lymphohistiocytosis (HLH) is a severe systemic hyperinflammatory syndrome characterized by dysregulated activation of macrophages and cytotoxic T lymphocytes, resulting in a cytokine storm and progressive multiorgan dysfunction [1,2]. HLH can be broadly classified into primary (familial) forms, typically presenting in childhood due to genetic defects in cytotoxic function, and secondary (acquired) forms, which occur in response to triggers such as infections, autoimmune diseases, or malignancies. In adults, HLH is almost invariably secondary, most commonly triggered by infections, autoimmune diseases, or malignancies [2]. Among malignancy-associated cases, hematologic neoplasms predominate, whereas solid tumors account for a minority [3,4].

Infectious triggers frequently include viral infections such as Epstein-Barr virus and cytomegalovirus, as well as bacterial, fungal, and parasitic infections. Autoimmune conditions, particularly systemic lupus erythematosus and adult-onset Still’s disease, are also well-recognized causes of secondary HLH, reflecting the broad spectrum of immune dysregulation underlying this syndrome.

The HLH-2004 diagnostic criteria, although widely used, were developed for pediatric populations and have not been formally validated in adults. Their sensitivity in adult populations is therefore limited. The HScore has emerged as a more appropriate diagnostic tool in this setting, providing a weighted probability of HLH based on clinical and laboratory parameters, with reported sensitivity of approximately 93-100% and specificity of 86-94% at commonly used thresholds (approximately 168-169).

The diagnosis of HLH in adults is particularly challenging due to its nonspecific clinical presentation, frequently mimicking severe infection or sepsis. This overlap contributes to delays in identifying the underlying cause, especially when HLH represents the initial manifestation of an occult malignancy. Diffuse gastric adenocarcinoma, particularly the signet ring cell subtype, is associated with aggressive biological behavior, early dissemination, and poor prognosis [5]. Although rare, HLH has been described in association with gastric malignancy, typically in advanced stages [6,7].

## Case presentation

A 38-year-old male, originally from Brazil and residing in Portugal, with no known past medical history, presented to the emergency department on November 4, 2025. He denied tobacco use, alcohol consumption, and illicit drug use. He denied recent travel, contact with animals, or other relevant epidemiological exposures. He reported prior self-administration of intramuscular testosterone on a weekly basis, initiated in early 2025 due to progressive weight loss, and discontinued approximately three months before presentation. Family history was unremarkable for malignancy or hematologic disease.

The patient described a three-month history of progressive, unintentional weight loss of approximately 12 kilograms. In the two weeks prior to admission, he developed recurrent high-grade fever (up to 39°C), associated with chills and drenching night sweats. He also reported severe and persistent lower back pain over the preceding two months, prompting multiple emergency department visits where only symptomatic treatment had been provided. At presentation, he had new-onset constipation and urinary retention, requiring bladder catheterization.

On physical examination, the patient appeared markedly cachectic and pale, with mild dehydration. Vital signs revealed a temperature of 38°C, heart rate of 105 beats per minute, and blood pressure of 122/70 mmHg. Abdominal examination demonstrated hepatomegaly palpable 2-3 cm below the right costal margin, without clinically evident splenomegaly. A left supraclavicular lymph node was palpable.

Initial laboratory evaluation (Table 1) revealed severe anemia (hemoglobin 4.5 g/dL), thrombocytopenia (43,000/µL), and leukocytosis with neutrophilia (13,300/µL; neutrophils 10,640/µL). Biochemical analysis demonstrated elevated lactate dehydrogenase (1,450 U/L), marked hyperferritinemia (19,710 ng/mL), hypertriglyceridemia (269 mg/dL), mild hepatic cytolysis (aspartate aminotransferase (AST) 61 U/L and alanine aminotransferase (ALT) 95 U/L), elevated cholestatic enzymes (gamma-glutamyl transferase (GGT) 432 U/L and alkaline phosphatase (ALP) 821 U/L), and hypoalbuminemia (2.8 g/dL). Haptoglobin was undetectable, raising suspicion of a hemolytic component.

**Table 1:** Laboratory findings at admission.

Parameter	Value	Reference Range
Hemoglobin	4.5 g/dL	13-17 g/dL
Platelet count	43,000/µL	150,000-400,000/µL
Leukocyte count	13,300/µL	4,000-11,000/µL
Neutrophil count	10,640/µL	2,000-7,500/µL
Ferritin	19,710 ng/mL	30-400 ng/mL
Lactate dehydrogenase (LDH)	1,450 U/L	135-225 U/L
Triglycerides	269 mg/dL	<150 mg/dL
Aspartate aminotransferase (AST)	61 U/L	<40 U/L
Alanine aminotransferase (ALT)	95 U/L	<41 U/L
Gamma-glutamyl transferase (GGT)	432 U/L	<60 U/L
Alkaline phosphatase (ALP)	821 U/L	40-130 U/L
Albumin	2.8 g/dL	3.5-5.0 g/dL
Haptoglobin	Undetectable	30-200 mg/dL

Peripheral blood smear demonstrated a leukoerythroblastic picture with schistocytes and circulating erythroblasts, suggestive of bone marrow infiltration.

An extensive infectious workup was performed, including serological testing for HIV, hepatitis B and C, Epstein-Barr virus, cytomegalovirus, syphilis, *Brucella*, *Coxiella burnetii*, and *Mycoplasma pneumoniae*, all negative for active infection. Blood and urine cultures were obtained at admission and remained negative throughout hospitalization.

Given the combination of persistent fever, bicytopenia, hyperferritinemia, hypertriglyceridemia, and hepatomegaly, an HScore was calculated (Table 2), indicating an 80-88% probability of HLH [8].

**Table 2:** HScore calculation for the diagnosis of hemophagocytic lymphohistiocytosis (HLH). A total HScore of 193 corresponds to an estimated probability of HLH of approximately 80-88%. AST: aspartate aminotransferase

Parameter	Patient Finding	Points
Known underlying immunosuppression	No	0
Temperature ≥38.4°C	Yes	33
Organomegaly (hepatomegaly)	Yes	23
Cytopenias (≥2 lineages)	Yes (anemia + thrombocytopenia)	24
Ferritin >6000 ng/mL	Yes (19,710 ng/mL)	50
Triglycerides >3 mmol/L (~265 mg/dL)	Yes (269 mg/dL)	44
AST >30 U/L	Yes (61 U/L)	19
Fibrinogen	Not available (not measured)	0
Hemophagocytosis in bone marrow	Not demonstrated on bone marrow biopsy	0
Total HScore	-	193

A bone marrow biopsy was performed; however, the sample was non-diagnostic due to extensive coagulative necrosis and absence of viable cellularity. Immunohistochemical staining (CKAE1/AE3 and CK8/18) demonstrated positivity in necrotic cells, strongly suggesting metastatic epithelial infiltration in the appropriate clinical context.

**Figure 1:**
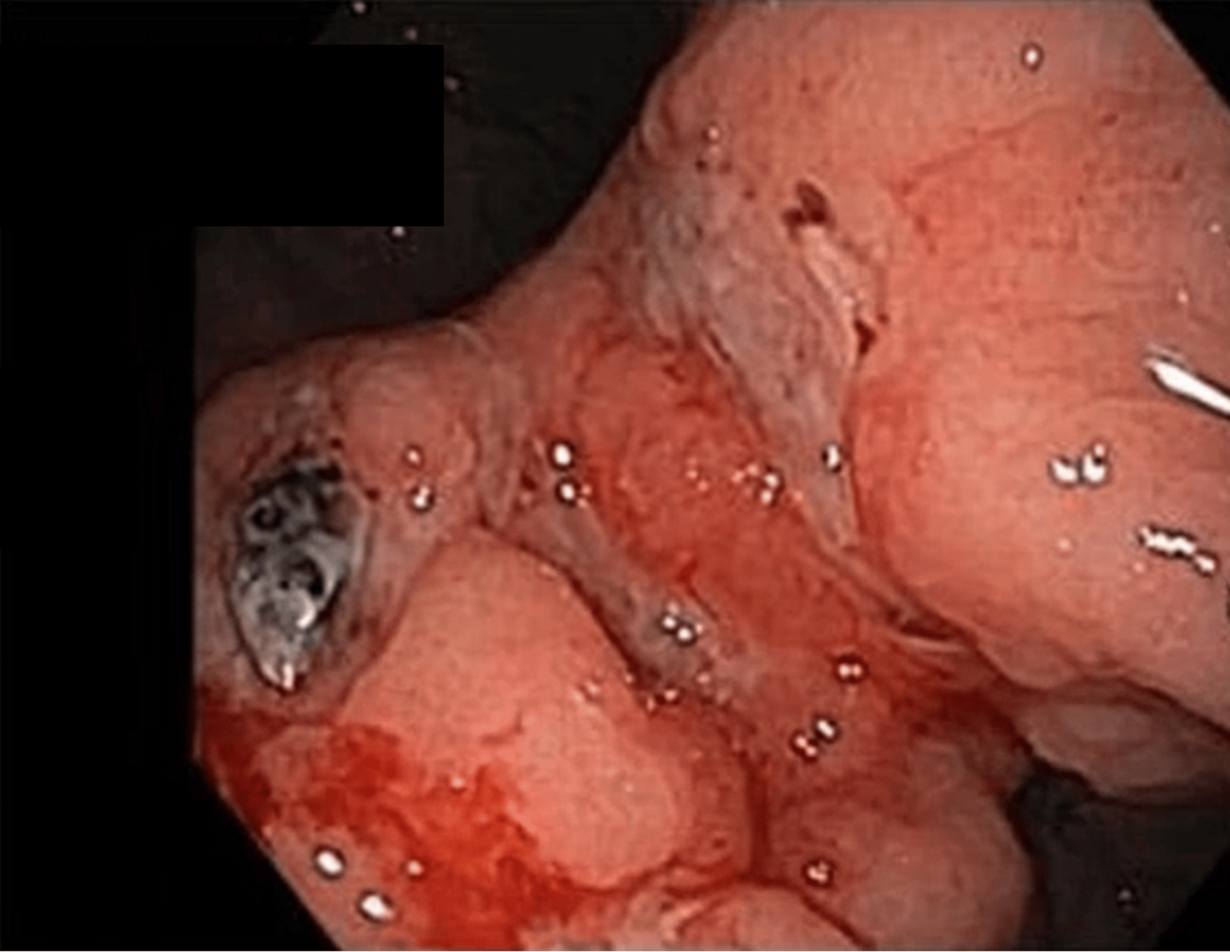
Upper gastrointestinal endoscopy showing large gastric ulcer.

**Figure 2:**
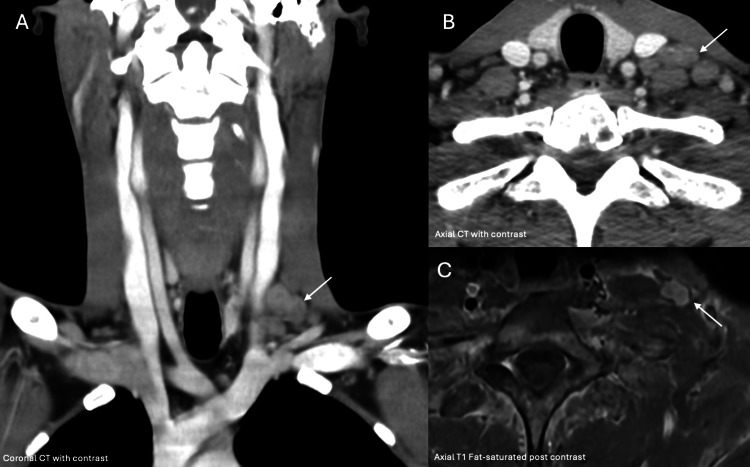
Contrast-enhanced neck CT (A, B) and cervical MRI (C) demonstrating a left supraclavicular nodal conglomerate, including involvement of Virchow’s node (arrows).

**Figure 3:**
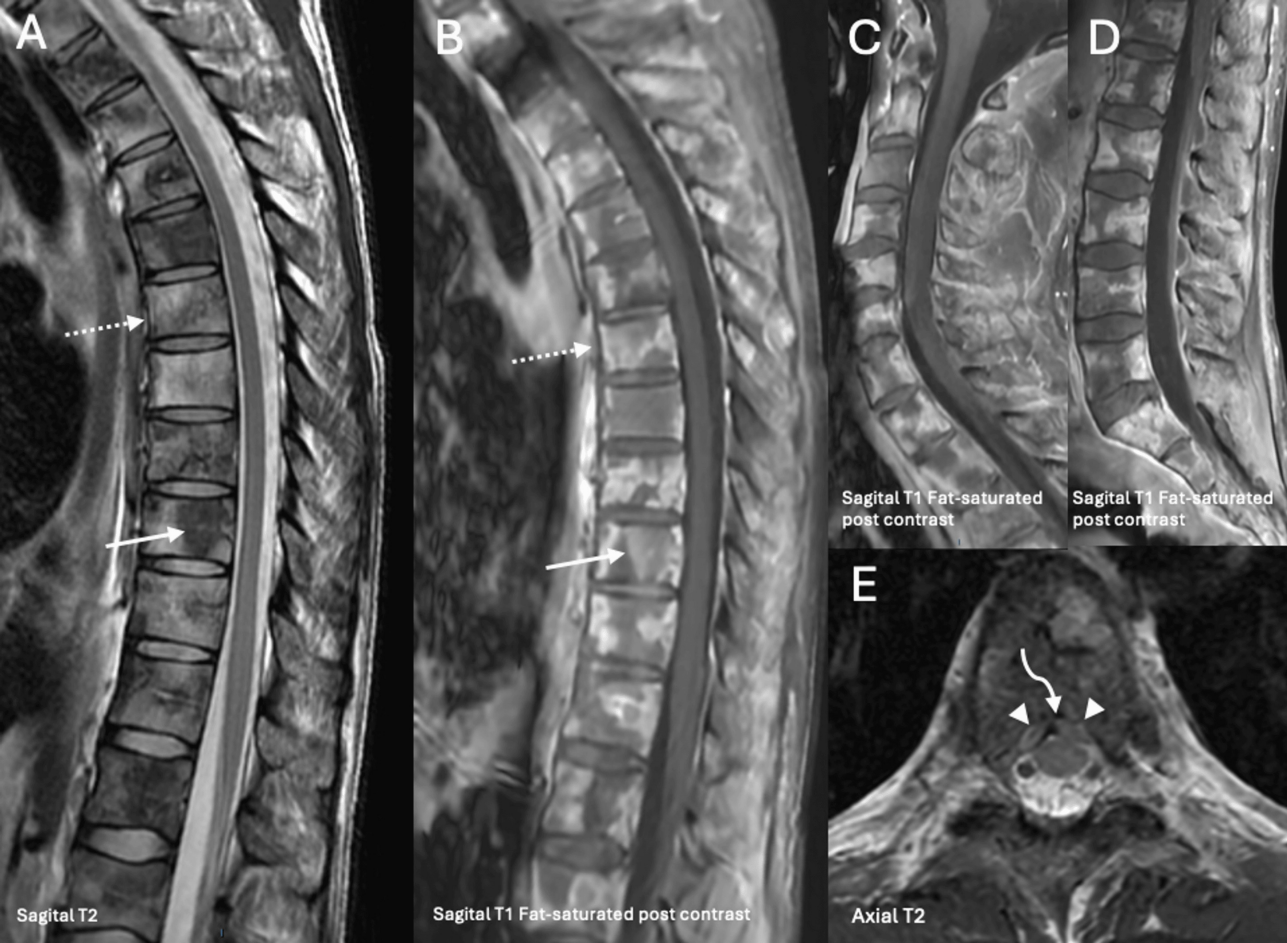
MRI showing heterogeneous signal throughout the cervical (C), thoracic (A, B), and lumbar spine (D) on pre- (A) and post-contrast sequences (B, C, and D), consistent with mixed sclerotic (white arrow) and lytic (dashed arrow) osseous metastatic disease. At the T6 level (E), anterior epidural metastatic extension demonstrates the “draped curtain sign” (white arrows), whereby posterior extension of a vertebral body lesion displaces the posterior longitudinal ligament. The characteristic contour reflects relative preservation of the medial meningovertebral ligament (curved arrow), whose firm midline attachment tethers the lesion centrally, resulting in a bilobulated intracanalicular appearance on axial images.

The diagnostic workup followed a sequential approach. Computed tomography (CT) of the chest, abdomen, and pelvis was performed on November 4, demonstrating hepatomegaly without focal hepatic lesions, no evidence of intra-abdominal masses, no significant splenomegaly or ascites, and no clear primary intra-abdominal tumor identified on initial imaging. This was followed by upper gastrointestinal endoscopy (Figure 1) that revealed multiple large gastric ulcers involving the antrum and incisura angularis and transthoracic echocardiography on November 7, bronchoscopy on November 10, and cervical CT with spinal magnetic resonance imaging (MRI) on November 13 (Figures 2-3).

Imaging (CT and MRI) revealed a left supraclavicular nodal conglomerate consistent with Virchow’s node (Figure 2). Spinal MRI demonstrated diffuse bone marrow infiltration with anterior epidural extension at the D6 level (Figure 3). Upper gastrointestinal endoscopy revealed multiple large gastric ulcers involving the antrum and incisura angularis (Figure 3).

Histopathological confirmation obtained on November 19 demonstrated diffuse gastric adenocarcinoma composed of poorly cohesive cells with signet ring morphology, with subsequent molecular characterization completed on November 28. Immunohistochemical evaluation demonstrated preserved expression of mismatch repair proteins (pMMR), PD-L1 expression with a combined positive score (CPS) of 5, HER2-negative status confirmed by in situ hybridization, and claudin 18 (CLDN18) positivity.

HLH was considered secondary to the underlying malignancy. Immunomodulatory therapy with anakinra (200 mg every eight hours) was initiated. Cytotoxic therapy with etoposide was not administered due to the absence of an initial confirmed malignant diagnosis, the need to exclude active infection, and persistent severe thrombocytopenia precluding safe administration.

Despite supportive care, including transfusion support, the patient experienced progressive clinical and laboratory deterioration, with persistent fever, worsening cytopenias, and multiorgan dysfunction. He died on December 2, 2025, 28 days after admission, before initiation of cancer-directed therapy due to rapid deterioration.

The clinical course is summarized in Table 3.

**Table 3:** Timeline of clinical presentation, diagnostic workup, and disease progression.

Date	Event
November 4, 2025	Presentation to the emergency department with fever, back pain, and cytopenias
November 4, 2025	CT of the chest, abdomen, and pelvis performed
November 7, 2025	Upper gastrointestinal endoscopy and echocardiography performed
November 10, 2025	Bronchoscopy performed
November 13, 2025	Cervical CT and spinal MRI performed
November 19, 2025	Histopathological diagnosis of gastric adenocarcinoma confirmed
November 28, 2025	Molecular characterization completed
December 2, 2025	Death due to multiorgan failure, 28 days after admission

## Discussion

HLH is characterized by uncontrolled immune activation leading to hypercytokinemia and multiorgan dysfunction [1,2]. In adults, secondary HLH is most frequently associated with infections or hematologic malignancies, while solid tumors represent a less common cause [3,4]. Malignancy-associated HLH has been extensively described in hematologic cancers, particularly T-cell and NK-cell lymphomas, but remains rare in the context of solid tumors, accounting for only a small proportion of reported cases [3,4,9]. When present, it is typically associated with advanced disease and carries a poor prognosis.

In the present case, diffuse gastric adenocarcinoma with extensive bone marrow infiltration likely played a central role in triggering HLH. Bone marrow involvement disrupts hematopoiesis and may contribute to hemophagocytic activation through tumor-driven cytokine dysregulation [2,6]. The leukoerythroblastic blood picture and the non-diagnostic but immunohistochemically suggestive bone marrow biopsy findings supported this mechanism. Furthermore, the presence of Virchow’s node, diffuse skeletal involvement, and epidural extension at diagnosis reflects a highly aggressive disease phenotype with early dissemination.

The combination of severe lower back pain and new-onset urinary retention represented important clinical red flags suggestive of spinal involvement, later confirmed by imaging as epidural extension of metastatic disease. The history of significant weight loss and recurrent fever was consistent with advanced malignancy; however, this clinical picture may have been partially masked by prior anabolic steroid use, which can preserve lean body mass and attenuate the overt manifestations of cancer-related cachexia. This highlights a clinically relevant but underrecognized confounding factor, particularly in younger patients, in whom exogenous androgen use may obscure the typical constitutional features of advanced malignancy and delay diagnostic suspicion.

This case also illustrates the well-recognized therapeutic paradox of malignancy-associated HLH, in which urgent control of the hyperinflammatory state is required, yet definitive treatment depends on addressing the underlying malignancy. In many cases, including the present one, severe cytopenias and rapid clinical deterioration preclude the initiation of systemic chemotherapy, ultimately determining prognosis.

The therapeutic approach prioritized cytokine-directed immunomodulation with anakinra. Anakinra is an interleukin-1 receptor antagonist that targets a central mediator of the hyperinflammatory cascade in HLH. By inhibiting IL-1 signaling, it may attenuate cytokine-driven immune dysregulation and has emerged as a therapeutic option in secondary HLH and macrophage activation syndrome-like conditions, particularly when standard cytotoxic therapy is contraindicated or not immediately feasible [10]. In critically ill patients, intravenous administration may offer more predictable pharmacokinetics compared to subcutaneous dosing, although robust comparative data remain limited. Given the absence of an initial confirmed malignant diagnosis and the need to exclude active infection, cytotoxic therapy with etoposide, as recommended in HLH protocols, was deferred [2]. Furthermore, persistent severe thrombocytopenia precluded its safe administration.

The tumor molecular profile (pMMR, PD-L1 CPS 5, HER2-negative, CLDN18-positive) would support standard first-line systemic therapy with platinum-fluoropyrimidine chemotherapy combined with immunotherapy, or consideration of CLDN18-targeted therapy [5]. In the current era of precision oncology, CLDN18-positive tumors may benefit from targeted agents such as zolbetuximab; however, in this case, the patient’s clinical condition precluded access to such therapies.

Overall, this case underscores the importance of early recognition of malignancy-associated HLH and the need for rapid multidisciplinary evaluation. Timely identification of the underlying malignancy is critical to preserve a therapeutic window; however, as illustrated here, the profound hematologic instability induced by HLH may itself become the principal barrier to disease-modifying treatment.

## Conclusions

HLH may represent the initial manifestation of advanced solid tumors, including diffuse gastric signet ring cell adenocarcinoma. Its clinical overlap with severe infection frequently leads to diagnostic delay and poor outcomes.

Early integration of oncologic evaluation in unexplained HLH is essential. This case highlights the critical therapeutic paradox in malignancy-associated HLH, in which the hyperinflammatory state may preclude timely initiation of the only treatment capable of addressing the underlying malignancy. Multidisciplinary management is critical to optimize the likelihood of initiating cancer-directed therapy, which remains the only intervention capable of altering prognosis.
